# Revenue Differences Between Top-Selling Small-Molecule Drugs and Biologics in Medicare

**DOI:** 10.1001/jamahealthforum.2025.4720

**Published:** 2025-10-17

**Authors:** Matthew Vogel, William B. Feldman, Zander Cowan, Benjamin N. Rome, Amitabh Chandra, Aaron S. Kesselheim, Olivier J. Wouters

**Affiliations:** 1 John F. Kennedy School of Government, Harvard University, Cambridge, Massachusetts; 2 Division of Pharmacoepidemiology and Pharmacoeconomics, Department of Medicine, Brigham and Women’s Hospital and Harvard Medical School, Boston, Massachusetts; 3 Division of Pulmonary and Critical Care Medicine, Department of Medicine, Brigham and Women’s Hospital and Harvard Medical School, Boston, Massachusetts; 4 Harvard Business School, Boston, Massachusetts; 5 Department of Health Services, Policy and Practice, Brown University School of Public Health, Providence, Rhode Island

## Abstract

This study compares the revenues earned by manufacturers 9 vs 13 years following US Food and Drug Administration approval for negotiation-eligible products from 2012 to 2022.

## Introduction

The Inflation Reduction Act (IRA) requires Medicare to negotiate prices for certain brand-name drugs with gross annual Medicare spending exceeding $200 million. Small-molecule drugs are exempt from negotiation for 9 years following US Food and Drug Administration approval and biologics for 13 years. The pharmaceutical industry, some members of Congress, and the Trump administration have argued that this difference prioritizes the development of biologics over small-molecule drugs and have proposed aligning the initial eligibility periods for both at 13 years.^1,2^ To inform these policy discussions, we compared the revenues earned by manufacturers after 9 vs 13 years on negotiation-eligible products from 2012 to 2022.

## Methods

We retrospectively applied the IRA’s eligibility criteria to Medicare drugs from 2012 to 2022 using the publicly available Medicare drug-spending dashboard.^3^ Following Medicare’s IRA implementation guidance,^4^ we aggregated spending by active moiety or ingredient and identified products meeting the $200 million spending threshold. Using previously described methods,^5^ we excluded products exempt from negotiation (eMethods in Supplement 1).

We obtained 1986-2024 annual US and global sales revenue (net of rebates and discounts) for each negotiation-eligible product from Evaluate Pharma, a third-party data provider (eMethods in Supplement 1). We compared median revenues for biologics and small-molecule drugs using 2-tailed Mann-Whitney U tests (α = .05). We evaluated the economic value of the first 13 years of US and global revenue using a 10.5% discount rate to account for the cost of capital.^1,2,5^ Revenues were inflation adjusted to 2024 dollars.

Institutional review board approval was not required for this study, as no data were collected from human participants. The study followed STROBE reporting guidelines.

## Results

**Figure 1.**
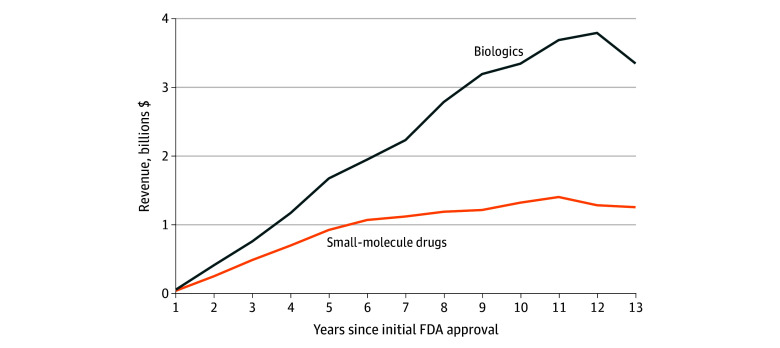
Median Annual Global Revenues for Products Eligible for Medicare Price Negotiation FDA indicates the US Food and Drug Administration.

From 2012 to 2022, 153 products (122 small-molecule drugs and 31 biologics) met IRA eligibility requirements in at least 1 year and had at least 13 years of revenue data available. Median (IQR) peak annual global revenue was higher for biologics ($3.8 billion [$2.3-$7.3 billion] in year 12) than for small-molecule drugs ($1.4 billion [$0.7-$2.8 billion] in year 11) (*P* < .001; Figure 1).

**Figure 2.**
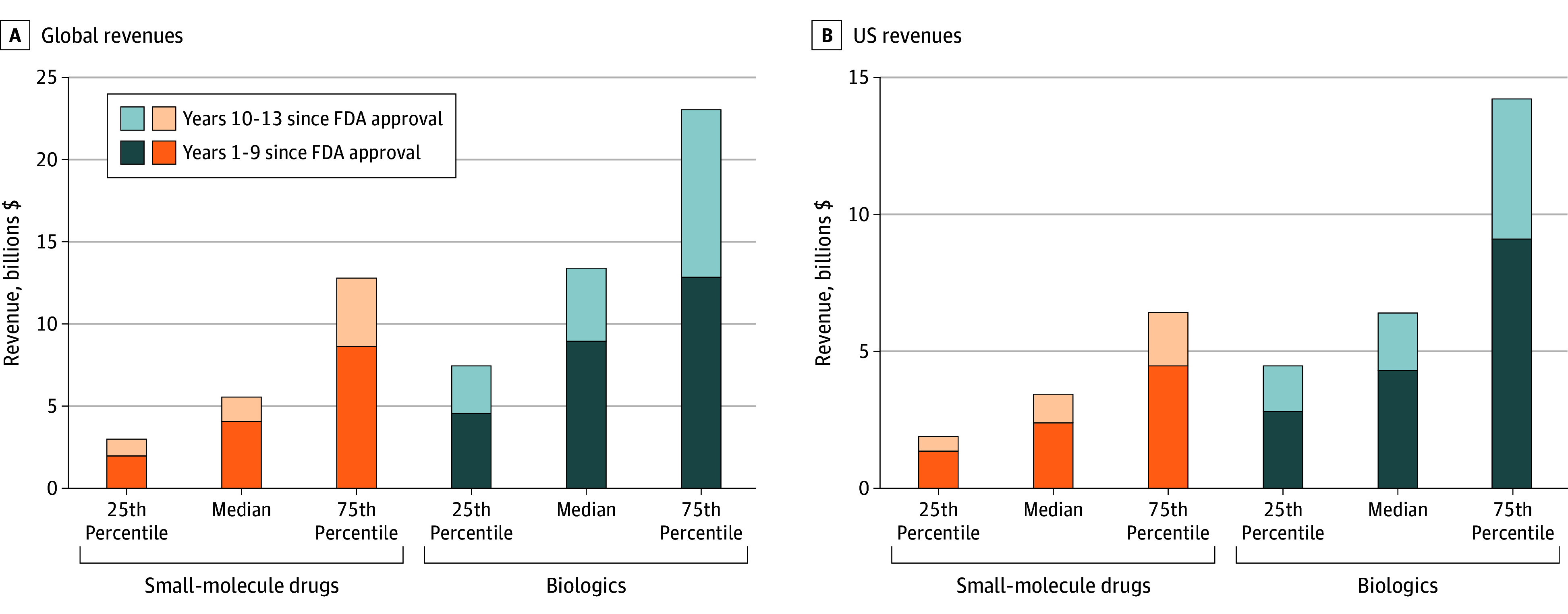
Economic Value of Global and US Revenues for Top-Selling Biologics and Small-Molecule Drugs FDA indicates the US Food and Drug Administration.

After 9 years, the median (IQR) economic value of global revenues was $4.1 billion ($2.0-$8.6 billion) for small-molecule drugs and $9.0 billion ($4.5-$12.8 billion) for biologics; by year 13, these values increased to $5.5 billion ($3.0-$12.8 billion) and $13.4 billion ($7.4-$23.0 billion), respectively (Figure 2). The share of year 1 to 13 economic value earned in years 10 to 13 was 27% for small-molecule drugs and 33% for biologics. For US revenues, the median (IQR) economic value after 9 years was $2.4 billion ($1.3-$4.5 billion) for small-molecule drugs and $4.3 billion ($2.8-$9.1 billion) for biologics; after 13 years it was $3.4 billion ($2.0-$6.8 billion) and $6.1 billion ($4.6-$13.6 billion), respectively (Figure 2).

## Discussion

In this study, among drugs that met Medicare price negotiation eligibility criteria from 2012 to 2022, biologics generated $4.9 billion more in cumulative revenue than small-molecule drugs over 9 years on the market and $7.9 billion more over 13 years. The gap between biologics and small-molecule drugs was larger than reported in previous studies that included less commercially successful products than those eligible for price negotiation.^1,2^ A limitation of this study is that revenues were analyzed retrospectively and do not reflect potential impacts of the IRA on development, commercialization, and pricing decisions, or the timing of generic or biosimilar entry. Additionally, this study does not include an analysis of the relative therapeutic value of different drug modalities.

A prior analysis found higher revenues and clinical trial success rates for biologics than small-molecule drugs and comparable development costs.^1^ The present study further supports the conclusion that biologics, on average, have generated substantially higher revenues than small-molecule drugs by year 9. Implementing negotiated prices later for biologics than for small-molecule drugs may exacerbate this difference, influence industry decisions about which drugs to develop, and limit potential savings from price negotiations. Future research should examine drug revenues alongside development costs and operating expenses to inform optimal timelines for price negotiation.

## Notes

Supplement 1.
eMethods

Supplement 2.
Data Sharing Statement
