# Supplementary material for: Revenue Differences Between Top-Selling Small-Molecule Drugs and Biologics in Medicare
Source: JAMA Health Forum. 2025 Oct 17;6(10):e254720. doi: 10.1001/jamahealthforum.2025.4720 (PMC12534848; doi:10.1001/jamahealthforum.2025.4720)
Supplement: Supplement 1. — eMethods [file jamahealthforum-e254720-s001.pdf]

Supplemental Online Content

Vogel M, Feldman WB, Cowan Z, et al. Revenue differences between top-selling small-molecule drugs and biologics in Medicare. *JAMA Health Forum*. 2025;6(10):e254720. doi:10.1001/jamahealthforum.2025.4720

eMethods

This supplemental material has been provided by the authors to give readers additional information about their work.

© 2025 Vogel M et al. *JAMA Health Forum*.

eMethods

1. Identifying Top-Selling Drugs

(a) *Spending Threshold*: Drugs with combined gross annual Medicare Part B and D spending exceeding $200 million are eligible for price negotiation under the Inflation Reduction Act (IRA). To identify drugs meeting the statutory spending requirement, we used the Medicare Drug Spending Dashboards to obtain Medicare Part B and D spending data from 2012-2022 (all the years for which data were available).[1,2] Because the $200 million threshold is denominated in 2022 dollars, we converted Medicare spending in each year to 2022 dollars using the US Department of Labor's Consumer Price Index (All Urban Consumers) as specified in the statute.[3,4]

(b) *Aggregating Spending Across Products*: The IRA requires the Centers for Medicare and Medicaid Services (CMS) to aggregate spending on all dosages and formulations of a 'drug' into a single sum. While the statute does not explicitly define a drug, it grants CMS discretion in determining this definition.[5] CMS has issued official implementation guidance, the most recent of which was published in November 2024, specifying its approach.[6] For the purposes of IRA eligibility, CMS defines a single drug to include any product made by the same company and containing the same active moiety or active ingredient. Spending on such products is aggregated regardless of whether they are marketed under the same or separate US Food and Drug Administration (FDA) applications. For example, Novo Nordisk's semaglutide products (Ozempic, Rybelsus, and Wegovy) were treated as a single product.

Where relevant, the total amount includes spending on authorized generics (i.e., generic versions of a drug marketed under the brand-name product's FDA approval), which can be marketed by the brand-name manufacturer (originator) or a licensed third party. To identify active moieties and ingredients, we used the Drugs@FDA and FDA's Structured Product Labeling databases.[7,8]

Following current CMS guidance, we treated fixed-dose combinations as distinct drugs, separate from other drugs containing the same active moieties or ingredients. In May 2025, CMS released a draft proposal that would revise this policy in the future. Under the proposal, fixed-dose combinations would not be considered distinct drugs if an additional active moiety or ingredient is "not biologically active against the disease state(s) the drug is indicated for and thus does not result in a clinically meaningful difference."[9] The proposed exception is likely intended to address concerns that reformulations, such as those that add hyaluronidase for subcutaneous injection, might defer price negotiation for several top-selling Medicare drugs. Although hyaluronidase is technically an active ingredient, in these cases it functions only as an excipient.[10] CMS is currently soliciting comments on the proposed exception. Following the release of the new proposal, we reviewed the historical spending data and did not identify any additional fixed-dose combination products that would apply for price negotiation under a plausible interpretation of the proposed exception.

© 2025 Vogel M et al. *JAMA Health Forum*.

(c) *Differences Between Part D and Part B*: Medicare maintains separate drug spending dashboards for Part D (retail pharmacy benefit) and Part B (hospital/inpatient benefit). The Part D dashboard includes both traditional fee-for-service spending and Medicare Advantage spending. The Part B dashboard only includes traditional fee-for-service drug spending (i.e., excludes Medicare Advantage). As a result, Part B drug spending is underreported. Recognizing that there is no publicly available data to bridge this gap, prior studies have primarily used unadjusted Part B spending dashboard data. An exception is Reitsma et al, [11] who "increased Part B spending to reflect the 40% of the Medicare population enrolled in a Medicare Advantage plan in 2020." They acknowledge, however, that "scaling Part B spending to account for Medicare Advantage spending assumed that spending patterns across Part B drugs are the same for fee-for-service and Medicare Advantage enrollees, which may be inaccurate."

Facing the same issue, we chose not to adjust the Part B data. Given known differences in patient characteristics, health needs, utilization management, and step therapy requirements between fee-for-service Medicare and Medicare Advantage,[12,13,14,15,16] it is unclear whether scaling would improve representativeness. In evaluating which methodology to apply, we determined that scaling Part B expenditures, as done by Reitsma et al, would add three drugs to our cohort (one biologic and two injectable small-molecule drugs) with negligible impact on results, as shown in the figure.

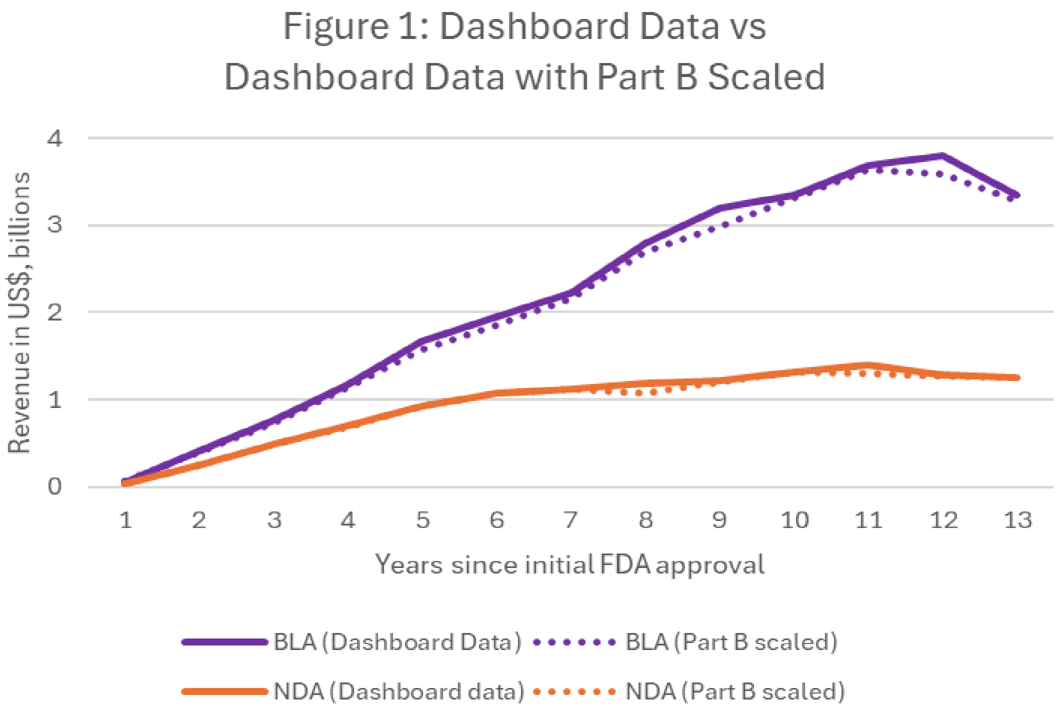

BLA indicates Biologics License Application; NDA, New Drug Application.

© 2025 Vogel M et al. *JAMA Health Forum*.

2. Applying Exclusions

To select our cohort, we first restricted the sample to top-selling products (i.e., those with more than $200 million in gross Medicare spending in any year from 2012-2022). To ensure that our cohort was focused on therapeutic products, we excluded diagnostics (e.g., imaging or contrast agents) and vaccines. The following products were removed from our cohort: Fluad Quad, Fluzone, Lexiscan, Prevnar 13, Shingrix, and Zostavax.

In addition to the $200 million minimum spending requirement, the IRA includes several statutory exemptions from price negotiation. Following the IRA's statutory criteria and the CMS implementation guidance, we assessed each product's eligibility for these exemptions as of December 31st in each year from 2012-2024:

(a) *Minimum Time Since Initial FDA Approval*: Negotiated prices cannot go into effect until 9 years after initial FDA approval for small-molecule drugs or 13 years for biologics. Because the IRA negotiation timeline includes a 2-year period between a drug being selected for negotiation and a negotiated price going into effect, a drug cannot be selected for negotiation earlier than 7 years after initial FDA approval (for small-molecule drugs) or 11 years (for biologics).[17]

    We used Drugs@FDA to identify the earliest FDA approval date for a drug (i.e., its active moiety or ingredient in an FDA-approved product marketed by the same manufacturer).[18]

(b) Generic or Biosimilar Competition: Drugs facing generic or biosimilar competition are exempt from negotiation. The CMS guidance indicates that competition against any dosage or formulation of a drug will exempt all dosages and formulations of that drug from negotiation.

    We identified generic versions using the FDA's Orange Book and biosimilar versions using the Purple Book.[19,20] As done in previous studies,[21,22] we used Medicaid data (updated through December 31, 2024) to determine the market entry dates of generics or biosimilars.[23] We also used this data to assess the applicability of the IRA's biosimilar delay rule, which allows for a delay of up to 2 years before a biologic can be selected for negotiation if the Secretary of Health and Human Services, at the request of a biosimilar manufacturer, determines that a biosimilar will be "imminently" marketed.[24]

(c) Products Derived from Human Plasma: The IRA exempts products derived from human plasma.[25]

    We used the FDA's Approved Blood Products website to identify exempt human plasma-derived products.[26]

© 2025 Vogel M et al. *JAMA Health Forum*.

(d) *Sole Orphan Drugs*: The IRA exempts drugs approved solely for the treatment of a single rare disease.[27]

We used the FDA's Orphan Drug Product designation database to identify drugs with a single orphan designation and, after consulting Drugs@FDA, excluded those with no indications outside its orphan designation.[28,29]

To be included in the analytical cohort, a product needed to have at least one year in which it satisfied the spending threshold but was not eligible for any exemption in that year.

We did not apply the IRA's fifth statutory exclusion for "small biotech" products due to its idiosyncratic and time-limited nature. Specifically, the IRA exempts products from negotiation for the years 2026, 2027, and 2028 (and only for those years) if they satisfy 3 criteria: (i) more than $200 million in gross Medicare spending in 2023, 2024, or 2025, (ii) gross Medicare spending in 2021 that was less than 1% of total gross Medicare spending, and (iii) gross Medicare revenues in 2021 that contributed more than 80% of the manufacturer's total gross Medicare revenues in 2021.[30] Based on 2021 Medicare Part B and Part D spending data, we identified 3 products that met those criteria: Jakafi (ruxolitinib, Incyte), Ingrezza (valbenazine, Neurocrine), and Cabometyx (cabozantinib, Exelixis).[31,32]

In addition to statutory exemptions, the IRA caps the number of drugs that can be selected for negotiation to 20 per year. However, we do not believe the cap to be material to the results of this study. In our cohort, we found no year from 2012-2022 in which more than 20 drugs that met the IRA eligibility criteria for the first time (inclusive of drugs that met the eligibility criteria but were not analyzed in our study due to incomplete revenue data). In historical trends continue in the future, we expect that within a few years all newly approved drugs will be selected for price negotiation in the first year in which they become eligible.

3. <u>Revenue Analysis</u>

For all drugs with >$200 million in gross Medicare spending in any year from 2012-2022, we obtained data on annual US and global revenues from Evaluate Pharma, a proprietary database used in previous peer-reviewed studies.[33,34,35,36,37,38,39]

Evaluate Pharma compiles product-level revenue data on an annual and quarterly basis from public securities filings (e.g., 10-K, 10-Q, and 20-F forms filed with the US Securities and Exchange Commission). Evaluate Pharma supplements this data with additional corporate disclosures made in press releases, annual reports, financial appendices, earnings call transcripts, and presentations at investor conferences. Evaluate Pharma converts values denominated in foreign currencies into US dollars and reports all data in nominal terms (i.e., not adjusted for inflation). We converted all values to 2024 dollars using the US Department of Labor's Consumer Price Index (All Urban Consumers).[40]

© 2025 Vogel M et al. *JAMA Health Forum*.

Construction of Revenue Curves

To conduct our revenue analysis, we aligned the reported annual revenues for each drug in our cohort such that "Year 1" corresponded to revenues reported in the year of the product's initial FDA approval.

We prepared revenue curves covering the first 13 years of net revenues for small-molecule drugs and biologics, corresponding to the 13-year period during which biologics are exempt from Medicare price negotiation. To generate these curves, we calculated the median value of net revenues for small-molecule drugs and biologics in each year. We reported the value and timing of the highest revenue year for each type of product.

Cumulative Revenues and Discount Rate for Economic Values

To calculate cumulative revenues, we discounted the year 1-13 revenues of each drug to generate economic (present) values. We reported the median present value of year 1-9 revenues and year 10-13 revenues for small-molecule drugs and biologics. Though some drugs have life cycles that extend beyond 13 years, we did not extend our analysis further given our focus on the distinction between small-molecule drugs and biologics at years 9 and 13.

Cumulative revenues were discounted to estimate economic values (i.e., present values). Because decisions about whether to pursue the development of a biologic or a small-molecule drug are made many years before a product generates revenue, we applied a 10.5% discount rate, consistent with previous studies of drug development.[41,42,43,44,45,46]

Other discount rates are plausible. A recent RAND study noted, "there has been much debate about the most appropriate approach to estimating the cost of capital, but there is no complete consensus."[47] In 2024, the Congressional Budget Office used an 8.6% discount rate in its model of the pharmaceutical industry.[48] A 2024 study by Sertkaya et al found that estimates of the cost of capital for the biopharmaceutical industry ranged from 8.1% to 14.5%; they used 11% in their analysis.[49] This aligns with a study by the consulting firms LEK, RAND, and SiRM, which cited a typical range of 8% to 12% in the literature and used a midpoint of 10%.[50] In all cases, the estimates reflect industry averages and are not specific to any individual drug.

© 2025 Vogel M et al. *JAMA Health Forum*.

eReferences

[1] US Centers for Medicare and Medicaid Services. Medicare Part B spending by drug. Accessed November 20, 2024. https://data.cms.gov/summary-statistics-on-use-and-payments/medicare-medicaid-spending-by-drug/medicare-part-b-spending-by-drug
[2] US Centers for Medicare and Medicaid Services. Medicare Part D spending by drug. Accessed November 20, 2024. https://data.cms.gov/summary-statistics-on-use-and-payments/medicare-medicaid-spending-by-drug/medicare-part-d-spending-by-drug
[3] Inflation Reduction Act, Sec. 1192(e)(3)(B)(ii).
[4] US Department of Labor CPI Inflation Calculator. Accessed March 22, 2025. https://www.bls.gov/data/inflation_calculator.htm.
[5] Inflation Reduction Act, Sec. 1192(d)(3)(B).
[6] US Centers for Medicare & Medicaid Services. Medicare Drug Price Negotiation Program: Final Guidance, Implementation of Sections 1191-1198 of the Social Security Act for Initial Price Applicability Year 2026, and Solicitation of Comments. Accessed November 20, 2024. https://www.cms.gov/files/document/medicare-drug-price-negotiation-program-final-guidance.pdf
[7] US Food and Drug Administration. Drugs@FDA: FDA-approved drugs. Accessed January 14, 2025. https://www.accessdata.fda.gov/scripts/cder/daf/index.cfm
[8] US Food and Drug Administration. Structured Product Labeling database. Accessed January 14, 2025. https://nctr-crs.fda.gov/fdalabel/ui/search
[9] US Centers for Medicare & Medicaid Services. Medicare Drug Price Negotiation Program: Draft Guidance, Implementation of Sections 1191 – 1198 of the Social Security Act for Initial Price Applicability Year 2028 and Manufacturer Effectuation of the Maximum Fair Price in 2026, 2027, and 2028. Accessed July 17, 2025. https://www.cms.gov/files/document/ipay-2028-draft-guidance.pdf.
[10] Kim J, Kesselheim AS, Cliff ERS, Rome BN. Medicare spending and use of subcutaneous biologic formulations with hyaluronidase. Oncologist. 2025 Jun 4;30(6):oyaf149.
[11] Reitsma MB, Dusetzina SB, Ballreich JM, Trujillo AJ, Mello MM. Examining Opportunities to Increase Savings From Medicare Price Negotiations. JAMA Intern Med. 2023 Jun 1;183(6):581-588.
[12] Xu JF, Anderson KE, Liu A, Miller BJ, Polsky D. Role of Patient Sorting in Avoidable Hospital Stays in Medicare Advantage vs Traditional Medicare. JAMA Health Forum. 2023 Nov 3;4(11):e233931.
[13] Nicholas LH, Polsky D, Darden M, Xu J, Anderson K, Meyers DJ. Is there an advantage? Considerations for researchers studying the effects of the type of Medicare coverage. Health Serv Res. 2024 Feb;59(1):e14264.
[14] Hyland MF, Sachs RM, Robillard L, Hayford TB, Bai G. Spending on and Use of Clinician-Administered Drugs in Medicare. JAMA Health Forum. 2023 Sep 1;4(9):e232941.
[15] Kyle MA, Keating NL. The Promise and Perils of Oncology Care in Medicare Advantage. JAMA Netw Open. 2024 Sep 3;7(9):e2434650.
[16] Anderson KE, Polsky D, Dy S, Sen AP. Prescribing of low- versus high-cost Part B drugs in Medicare Advantage and traditional Medicare. Health Serv Res. 2022 Jun;57(3):537-547.
[17] Inflation Reduction Act, Sec. 1192(e)(1).
[18] US Food and Drug Administration. Drugs@FDA: FDA-approved drugs. Accessed January 14, 2025. https://www.accessdata.fda.gov/scripts/cder/daf/index.cfm
[19] US Food and Drug Administration Orange Book: Approved Drug Products With Therapeutic Equivalence Evaluations. Accessed January 14, 2025. https://www.accessdata.fda.gov/scripts/cder/ob/default.cfm.
[20] US Food and Drug Administration. Purple Book: Lists of Licensed Biological Products with Reference Product Exclusivity and Biosimilarity or Interchangeability Evaluations. January 14, 2025. https://www.fda.gov/drugs/therapeutic-biologics-applications-bla/purple-book-lists-licensed-biological-products-reference-product-exclusivity-and-biosimilarity-or.
[21] Vogel M, Kakani P, Chandra A, Conti RM. Medicare price negotiation and pharmaceutical innovation following the Inflation Reduction Act. Nat Biotechnol. 2024 Mar;42(3):406-412.

© 2025 Vogel M et al. *JAMA Health Forum*.

[22] Rome BN, Lee CC, Kesselheim AS. Market Exclusivity Length for Drugs with New Generic or Biosimilar Competition, 2012–2018. Clinical Pharmacology and Therapeutics. 109(2),367-371 (2021).
[23] Centers for Medicare and Medicaid Services. Drug Products in the Medicaid Drug Rebate Program. Accessed January 14, 2025. https://data.medicaid.gov/dataset/0ad65fe5-3ad3-5d79-a3f9-7893ded7963a
[24] Inflation Reduction Act, Sec. 1192(f).
[25] Inflation Reduction Act, Sec. 1192(e)(3)(C).
[26] US Food and Drug Administration. Approved Blood Products. January 14, 2025. https://www.fda.gov/vaccines-blood-biologics/blood-blood-products/approved-blood-products
[27] Vogel M, Zhao O, Feldman WB, Chandra A, Kesselheim AS, Rome BN. Cost of Exempting Sole Orphan Drugs From Medicare Negotiation. JAMA Intern Med. 2024 Jan 1;184(1):63-69.
[28] Inflation Reduction Act, Sec. 1192(e)(3)(A).
[29] US Food and Drug Administration. Search Orphan Drug Designations and Approvals. January 14, 2025. https://www.accessdata.fda.gov/scripts/opdlisting/oopd/
[30] Inflation Reduction Act, Sec. 1192(d)(2).
[31] US Centers for Medicare and Medicaid Services. Medicare Part B spending by drug. Accessed November 20, 2024. https://data.cms.gov/summary-statistics-on-use-and-payments/medicare-medicaid-spending-by-drug/medicare-part-b-spending-by-drug
[32] US Centers for Medicare and Medicaid Services. Medicare Part D spending by drug. Accessed November 20, 2024. https://data.cms.gov/summary-statistics-on-use-and-payments/medicare-medicaid-spending-by-drug/medicare-part-d-spending-by-drug
[33] Mulcahy A, Rennane S, Schwam D, Dickerson R, Baker L, Shetty K. Use of Clinical Trial Characteristics to Estimate Costs of New Drug Development. JAMA Netw Open. 2025 Jan 2;8(1):e2453275.
[34] Chandra A, Drum J, Daly M, Mirsberger H, Spare S, Neumann U, Martin S, Kirson N. Comprehensive measurement of biopharmaceutical R&D investment. Nat Rev Drug Discov. 2024 Sep;23(9):652-653.
[35] Jentzsch V, Osipenko L, Scannell JW, Hickman JA. Costs and Causes of Oncology Drug Attrition With the Example of Insulin-Like Growth Factor-1 Receptor Inhibitors. JAMA Netw Open. 2023 Jul 3;6(7):e2324977
[36] Spring L, Demuren K, Ringel M, Wu J. First-in-class versus best-in-class: an update for new market dynamics. Nat Rev Drug Discov. 2023 Jul;22(7):531-532.
[37] Lee M, Ly H, Möller CC, Ringel MS. Innovation in Regulatory Science Is Meeting Evolution of Clinical Evidence Generation. Clin Pharmacol Ther. 2019 Apr;105(4):886-898.
[38] Moser J, Verdin P. Trial watch: Burgeoning oncology pipeline raises questions about sustainability. Nat Rev Drug Discov. 2018 Oct 1;17(10):698-699.
[39] Cha M, Rifai B, Sarraf P. Pharmaceutical forecasting: throwing darts? Nat Rev Drug Discov. 2013 Oct;12(10):737-8.
[40] US Department of Labor CPI Inflation Calculator. Accessed March 22, 2025. https://www.bls.gov/data/inflation_calculator.htm.
[41] Wouters OJ, Vogel M, Feldman WB, Beall RF, Kesselheim AS, Tu SS. Differential Legal Protections for Biologics vs Small-Molecule Drugs in the US. JAMA. 2024 Nov 25.
[42] Wouters OJ, Kesselheim AS, Kuha J, Luyten J. Sales Revenues for New Therapeutic Agents Approved by the United States Food and Drug Administration From 1995 to 2014. Value Health. 2024 Oct;27(10):1373-1381.
[43] Vogel M, Kakani P, Chandra A, Conti RM. Medicare price negotiation and pharmaceutical innovation following the Inflation Reduction Act. Nat Biotechnol. 2024 Mar;42(3):406-412.
[44] Wouters OJ, McKee M, Luyten J. Estimated Research and Development Investment Needed to Bring a New Medicine to Market, 2009-2018. JAMA. 2020 Mar 3;323(9):844-853.
[45] DiMasi JA, Grabowski HG, Hansen RW. Innovation in the pharmaceutical industry: New estimates of R&D costs. J Health Econ. 2016 May;47:20-33.
[46] Chandra A and Mazundar S. Biotech Asset Valuation Methods: A Practitioner's Guide. Journal of Investment Management. 2024:22(1):36-57.
[47] Sussex J, Davies C, Marciniak-Nuqui Z, Cabling M, Mestre-Ferrandiz J, Mulcahy A. Impact of Increasing Requirements for Research and Development (R&D) Cost Transparency. RAND Europe. 2023. https://www.rand.org/content/dam/rand/pubs/research_reports/RRA2500/RRA2519-1/RAND_RRA2519-1.pdf. Accessed: July 17, 2025.

© 2025 Vogel M et al. *JAMA Health Forum*.

[48] Adams C. CBO's Model of Drug Development: Ongoing Updates. Congressional Budget Office. 2024 November 22. https://www.cbo.gov/system/files/2024-11/60771-Drug-Innovation-Model.pdf. Accessed: July 17, 2025.

[49] Sertkaya A, Beleche T, Jessup A, Sommers BD. Costs of Drug Development and Research and Development Intensity in the US, 2000-2018. JAMA Netw Open. 2024 Jun 3;7(6):e2415445.

[50] The Financial Ecosystem of Pharmaceutical R&D: An Evidence Base to Inform Further Dialogue. LEK, RAND Europe, SiRM. February 2022. https://www.lek.com/sites/default/files/PDFs/financial-ecosystem-rd.pdf. Accessed: July 17, 2025.

© 2025 Vogel M et al. *JAMA Health Forum*.
