# Supplementary material for: Revenue Differences Between Top-Selling Small-Molecule Drugs and Biologics in Medicare
Source: JAMA Health Forum. 2025 Oct 17;6(10):e254720. doi: 10.1001/jamahealthforum.2025.4720 (PMC12534848; doi:10.1001/jamahealthforum.2025.4720)
Supplement: Supplement 2. — Data Sharing Statement [file jamahealthforum-e254720-s002.pdf]

Data Sharing Statement

Vogel. Revenue Differences Between Top-Selling Small-Molecule Drugs and Biologics in Medicare. *JAMA Health Forum*. Published October 17, 2025.
doi:10.1001/jamahealthforum.2025.4720

Data
Data available: No

Additional Information
Explanation for why data not available: Data on sales revenues were sourced from Evaluate Pharma. All other data were obtained from publicly available sources.
